# Heat Acclimation Following Heat Acclimatization Elicits Additional Physiological Improvements in Male Endurance Athletes

**DOI:** 10.3390/ijerph18084366

**Published:** 2021-04-20

**Authors:** Courteney L. Benjamin, Yasuki Sekiguchi, Jeb F. Struder, Michael R. Szymanski, Ciara N. Manning, Andrew J. Grundstein, Elaine C. Lee, Robert A. Huggins, Lawrence E. Armstrong, Douglas J. Casa

**Affiliations:** 1 Department of Kinesiology, Korey Stringer Institute, University of Connecticut, Storrs, CT 06269, USA; yasuki.sekiguchi@uconn.edu (Y.S.); jeb.struder@uconn.edu (J.F.S.); michael.szymanski@uconn.edu (M.R.S.); ciara.manning@uconn.edu (C.N.M.); robert.huggins@uconn.edu (R.A.H.); uconnla@aim.com (L.E.A.); douglas.casa@uconn.edu (D.J.C.); 2 Department of Kinesiology, Samford University, Birmingham, AL 35229, USA; 3 Department of Geography, University of Georgia, Athens, GA 30602, USA; andrewg@uga.edu; 4 Human Performance Laboratory, Department of Kinesiology, University of Connecticut, Storrs, CT 06269, USA; elaine.c.lee@uconn.edu

**Keywords:** thermoregulation, heat mitigation, aerobic, heat tolerance, heat illness, training strategy

## Abstract

The purpose of this study was to assess the effectiveness of heat acclimatization (HAz) followed by heat acclimation (HA) on physiological adaptations. 25 male endurance athletes (age 36 ± 12 y, height 178.8 ± 6.39 cm, body mass 73.03 ± 8.97 kg, and VO_2peak_ 57.5 ± 7.0 mL·kg^−1^·min^−1^) completed HAz and HA. HAz was 3 months of self-directed summer training. In the laboratory, a 5-day HA prescribed exercise to target a hyperthermic zone (HZHA) of T_rec_ between 38.50 and 39.75 °C for 60 min. Exercise trials were 60 min of running (59% ± 2% VO_2peak_) in an environmental chamber (wet bulb globe temperature 29.53 ± 0.63 °C) and administered at: baseline, post-HAz, and post-HAz+HA. Measured variables included internal body temperature (T_rec_), heart rate (HR), and sweat rate (SR). Repeated measure ANOVAs and post hoc comparisons were used to assess statistically significant (*p* < 0.05) differences. T_rec_ was lower post-HAz+HA (38.03 ± 0.39 °C) than post-HAz (38.25 ± 0.42 °C, *p* = 0.009) and baseline (38.29 ± 0.37 °C, *p* = 0.005). There were no differences between baseline and post-HAz (*p* = 0.479) in T_rec_. HR was lower post-HAz (143 ± 12 bpm, *p* = 0.002) and post-HAz+HA (134 ± 11 bpm, *p* < 0.001) than baseline (138 ± 14 bpm). HR was lower post-HAz+HA than post-HAz (*p* = 0.013). SR was higher post-HAz+HA (1.93 ± 0.47 L·h^−1^) than post-HAz (1.76 ± 0.43 L·h^−1^, *p* = 0.027). Combination HAz and HA increased physiological outcomes above HAz. This method can be used to improve performance and safety in addition to HAz alone.

## 1. Introduction

Athletes from around the world travel to compete in hot weather conditions of which they are not accustomed to [1]. Heat acclimatization (HAz) is a heat mitigation strategy that is often proposed to athletes and coaches as a means of optimizing performance and preventing heat illness. The idea of HAz is simple: train outside in the heat to adapt. The implementation of this idea with athletes, however, is not as simple to effectively implement. For example, regional differences in environmental conditions, such as the mild summers experienced by many across the globe, make it difficult for athletes who are traveling to hotter venues to gain the full benefits of HAz needed prior to competition [2].

One solution to this problem is the use of heat acclimation (HA), which is the systematic process of repeated exposures to a thermally extreme environment that elicits positive physiological and perceptual adaptations [3] in an artificial environment. Typical responses following HA include lower heart rate (HR), internal body temperature, skin temperature (T_sk_), and sweat electrolyte concentration, and increased plasma volume and sweat rate (SR) [4]. The time course of HA adaptions are well-established, with SR typically being the adaptation that occurs at least 7–10 days into HA [4,5,6,7]. While this strategy is an effective solution, the cost and time constraints that are often placed on athletes and coaches make the recommended 7–14 day protocols difficult to achieve. There is no consensus on a universal, optimal protocol [7] for HA, HAz, or a combination of the two.

One way in which HAz may be advantageous to HA in most environmental laboratories is that HAz introduces radiant heat from the sun as a stimulus for physiological adaptation, but HAz may not provide an adequate thermal load (depending on training location and environmental conditions) for optimal thermoregulatory adaptations [8]. HA may produce greater physiological and performance outcomes than HAz, however, it can be difficult to balance normal sport training with an intense HA protocol for a long period of time [3]. Even amongst HA protocols, various methods have been utilized [7,9] and no consensus has been made as to the “optimal” approach.

The fixed-work rate method, that involves a steady exercise intensity, can elicit a thermal stress through an elevated internal body temperature. However, one limitation to this method is that if the workload is intense enough to drive internal body temperature up, the session must stop when the participant experiences volitional fatigue or the laboratory temperature cut-off point is reached, potentially limiting the duration that an individual is exercising in these conditions. Alternatively, if the intensity is too low, the drive to reach an elevated internal body temperature will lead to a long session duration, which is often not feasible for athletes when attempting to maintain an appropriate training balance [10]. Self-paced HA has also been examined in previous research, however, this method allows the athlete to self-select the intensity, which could result in a reduced thermal, cardiovascular, and relative work-load [7].

One of the most popular methods of HA is the isothermal approach, which involves continuously adjusting exercise intensity to maintain an internal body temperature of 38.50 °C [4]. One limitation to this method of HA is the variability in cardiovascular and workload responses between individuals. One of the largest limitations of this method is based on the overload principle of training and the fact that athletes often reach internal body temperatures well above this threshold [11]. A HA protocol that involves greater internal body temperatures may produce greater physiological and perceptual adaptations and there is a need to investigate such protocol.

Since HAz in milder climates may not induce the needed adaptations for hot weather competitions, HAz and HA may be prescribed together to optimize practicality and efficiency while improving physiological benefits associated with either alone, but little is known about combined protocols, especially with a more intense HA protocol. A previous study that investigated combining two forms of HA did not find evidence that this method was any more effective than a traditional HA protocol [12]. However, the length of the second, more intense portion of HA was only implemented for 3 days. This, in addition to the altogether low levels of hyperthermia that was achieved in this protocol, leaves speculation as to whether a longer and/or more intense protocol would have resulted in similar results. Time and practicality of implementation are often limiting factors when selecting the appropriate heat mitigation strategy in sport and military settings. A unique protocol that involves 5 days of HA following HAz could offer all of the physiological and perceptual benefits observed in 7–14 days of HA with less time required in an artificial laboratory, making the idea of HA implementation more appealing to athletes and coaches. Additionally, HAz is often already utilized in these settings and the addition of a short-term HA protocol could provide additional benefits that could lead to enhanced performance and safety. Upon successful implementation of a short-term HA protocol to supplement HAz, sport and military personnel may be more likely to adopt this heat mitigation strategy.

Therefore, the aim of this study was to assess the effectiveness of HAz followed by short-term HA on physiological and perceptual variables during steady-state exercise in the heat. We hypothesize that physiological and perceptual adaptations would be observed following HAz in an aerobically trained population, and that additional adaptations would follow a novel short-term HA protocol. Successful completion of this protocol and confirmation of these hypotheses would provide coaches, sports medicine professionals, and military personnel with an effective, practical training approach when preparing athletes and warfighters for exercise in hot environments.

## 2. Materials and Methods

**Figure 1:**
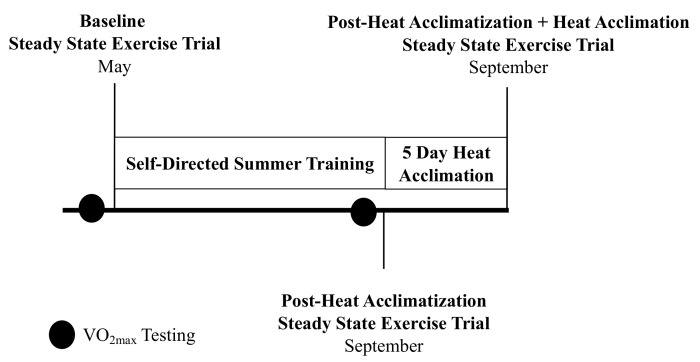
Study timeline. Baseline, post-heat acclimatization, and post-heat acclimation.

Twenty-five male endurance athletes were included in this study (age, 36 ± 12 y; height, 178.81 ± 6.39 cm; body mass, 73.03 ± 8.97 kg; and VO_2peak_ 57.48 ± 7.03 mL·kg^−1^·min^−1^). These participants were recruited from the local running and cycling community. This study was approved by the University of Connecticut institutional review board and all participants provided written informed consent. A within-participant longitudinal study design was utilized (Figure 1).

**Figure 2:**
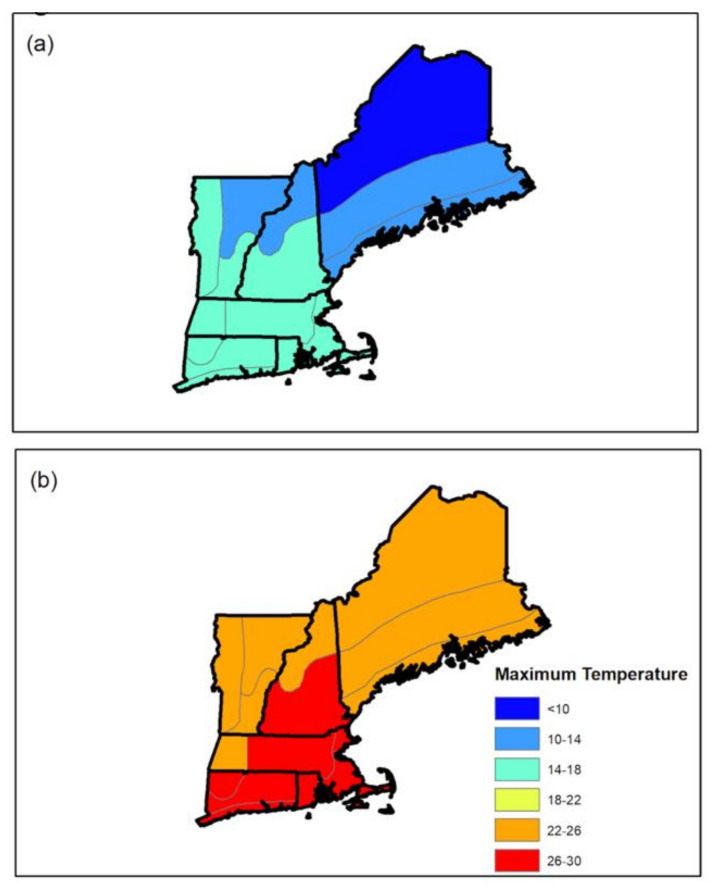
(**a**) Average maximum monthly ambient temperature in New England by climate division for April–May (baseline; unacclimatized) in 2019 and (**b**) average maximum monthly ambient temperature in New England by climate division for June–August (heat acclimatization) in 2019.

To assess physiological and perceptual adaptations, participants completed 60 min of steady state exercise (59% ± 2% vVO_2peak_) in an artificial environmental laboratory (ambient temperature (T_amb_) 35.11 ± 0.62 °C, relative humidity (RH) 47.61% ± 0.38%, wet bulb globe temperature (WBGT) 29.53 ± 0.63 °C, wind speed, 4.02 ± 0.12 mph) at three timepoints: baseline, post-HAz, and post-HAz+HA. All trials were performed on a motorized treadmill (T150; COSMED, Traunstein, Germany). These environmental conditions were chosen to reflect red flag conditions for physical activity [2]. The number of days between baseline and post-HAz were recorded (baseline and post-HAz, 109 ± 9 days). It was assumed that participants were unacclimatized at baseline, as all participants resided in the northeastern states of the United States and the environmental conditions were higher following the baseline trial (Figure 2) than prior to it.

Throughout the trials, physiological (HR, rectal temperature [T_rec_], and T_sk_) and perceptual (rating of perceived exertion (RPE), thermal sensation (TS), thirst, and fatigue) measures were recorded every five minutes. HR was measured with a chest strap (H10^®^, Polar Electro™, Kempele, Finland) and participants were instructed to insert a rectal probe 10 cm passed the anal sphincter for T_rec_ to be recorded (MP160; BIOPAC Systems Inc., Goleta, CA, USA). T_sk_ was measured on four sites (iButton; iButton Link LLC., Whitewater, WI, USA), including the thigh, chest, upper arm, and calf, and mean T_sk_ was calculated [13]. SR was calculated by taking the difference in nude body mass measurements assessed before and immediately post exercise, accounting for urinary losses. Sweat electrolyte concentration (sodium (Na^+^), potassium (K^+^), and chloride (Cl^−^)) was also assessed via the whole-body wash-down technique [14]. Participants were instructed to arrive to the laboratory euhydrated and this was confirmed with urine indices (urine specific gravity, 1.010 ± 0.008; and urine color, 2 ± 0) [15]. No fluid was provided throughout the 60 min of exercise.

Due to the longitudinal nature of this study, VO_2peak_ and vVO_2peak_ changes were assessed to ensure that there were no changes in aerobic fitness that could influence the physiological variables observed in the trials. VO_2peak_ and vVO_2peak_ was assessed prior to baseline and post-HAz. Participants were asked to don a HR monitor (H10^®^, Polar Electro™, Kempele, Finland) and complete a self-selected 5-min warm-up. Following warm-up, participants completed a graded maximal exercise test on a treadmill (T150; COSMED, Traunstein, Germany) at 2% grade to volitional exhaustion (TrueOne 2400, ParvoMedics, Sandy, UT, USA). Specifically, the speed on the treadmill increased by 1.0 or 0.5 miles per hour (mph) at the end of each 2-min stage until volitional exhaustion. The relative volume of oxygen recorded during the final completed stage was reported as the VO_2peak_ and the velocity at this stage was reported as the vVO_2peak_.

Following baseline trials, participants completed and recorded self-directed summer training between baseline and post-HAz. Participants utilized their own training devices (Garmin, *n* = 21 (Forerunner^®^ Fenix^®^ Vivoactive^®^ Garmin™ Ltd., Olathe, KS, USA); Polar H10 and Polar Beat application, *n* = 3 (H10^®^, Polar Electro™, Kempele, Finland)) [16]. In addition to these devices, 3 participants also utilized cycling computers to track their cycling training (Wahoo ELEMNT Bolt, *n* = 1 (ELEMNT Bolt, Wahoo Fitness^®^, Atlanta, GA, USA), Garmin Edge, *n* = 1 (Edge^®^, Garmin Ltd., Olathe, KS, USA), Bryton Rider 15, *n* = 1 (Rider 15^®^, Bryton™ Inc., Taipei City, Taiwan)). No training instruction was given during this period. Meteorological data from training sessions that were performed outside (with the exception of swimming) were extracted from the nearest available automated surface observing station (ASOS), within a mean distance of 16±11 km from the location of training. The location of training was determined by the GPS device and the latitude/longitude of that training session location was utilized to determine the closest weather station. Daytime WBGTs (7 a.m.–7 p.m.) were modeled using a Heat Stress Advisor software package (version 2005; Zunis Foundation, Tulsa, OK, USA; Coyle 2000) [17], which is designed to work with weather station data; nighttime WBGTs were computed using the Liljegren model with solar radiation set to zero [18]. Total distance, average HR, session duration, T_amb_, %RH, heat index, and WBGT were reported (Table 1). Indoor training was excluded from this analysis.

Following post-HAz, participants completed a five-day HA protocol in an artificial environmental laboratory (T_amb_, 38.67 ± 1.03 °C; %RH, 51.34 ± 2.42%; WBGT, 33.82 ± 1.20 °C; wind speed, 0 ± 0 mph). These environmental conditions were chosen to maximize the internal temperature response. Five HA sessions were completed within eight days and the number of days between each HA session and tests were recorded (post-HAz and HA^#1^, 4 ± 2 days; HA^#1^ and HA^#2^, 1 ± 1 day; HA^#2^ and HA^#3^, 2 ± 1 days; HA^#3^ and HA^#4^, 2 ± 1 days; HA^#4^ and HA^#5^, 1 ± 1 days; total number of HA days, 6 ± 1 days; HA^#5^ and post-HA, 3 ± 1 days). The HA sessions involved exercise to induce hyperthermia for 60 min and is termed hyperthermic zone HA (HZHA). Hyperthermia was defined as temperatures between 38.50 and 39.75 °C. In general, the exercise sessions began with a higher intensity of exercise (70% vVO_2peak_) and the intensity was adjusted throughout the session to allow the participant to experience hyperthermia for 60 min. Total T_rec_ and T_rec_ above 38.50 °C integral area under the curve (AUC) was calculated for each HA session (Table 2).

The sample size calculation was based on the second part of this two-part study in which participants were assigned to groups following HA. The calculation was performed in G*Power (version 3.1, Dusseldorf, Germany) and is based on the variability of internal body temperature between two groups from a heat training intervention in a previous study examining the effectiveness of heat exposures every five days following HA [19]. For a two-sided test with 0.05 alpha level and desired power level of 0.8 the estimated sample size would be 24 participants. Repeated measure ANOVAs were utilized to determine differences in physiological and perceptual outcomes between testing time points. For all analyses, in the presence of a significant Mauchly’s test of sphericity, greenhouse–Geisser correction was used. Pairwise differences were assessed post-hoc using LSD. Cohen’s d effect sizes (ES) were calculated to quantify the magnitude of pairwise differences. ES was interpreted according to the following thresholds: < 0.2 = trivial, 0.2–0.6 = small, 0.7–1.1 = moderate, 1.2–2.0 = large, and > 2.0 = very large [20]. Statistical significance was set at *p* < 0.05, a priori. Data are reported in text as mean ± standard deviation. All statistical analyses were completed using SPSS Statistics for Mac, version 25 (IBM Corp., Armonk, NY, USA).

## 3. Results

There were no differences in VO_2peak_^#1^ (57.9 ± 6.8 ML kg^−1^·min^−1^) and VO_2peak_^#2^ (59.7 ± 8.2, *p* = 0.67). There were also no differences in vVO_2peak_^#1^ (10.0 ± 0.5 mph) and vVO_2peak_^#2^ (10.0 ± 0.5 mph, *p* = 0.21). Self-directed summer training was recorded, and descriptive training and environmental data can be seen in Table 1. The average T_rec_ AUC experienced during HA was 216 ± 15 (°C·h^−1^). Descriptive data from HA can be seen in Table 2.

### 3.1. Heart Rate

Differences in physiological outcomes between baseline, post-HAz, and post-HAz+HA can be seen in Table 3. Significant main effects were observed in average HR (*p* < 0.001) and max HR (*p* < 0.001), but not resting HR (*p* = 0.67).

**Figure 3:**
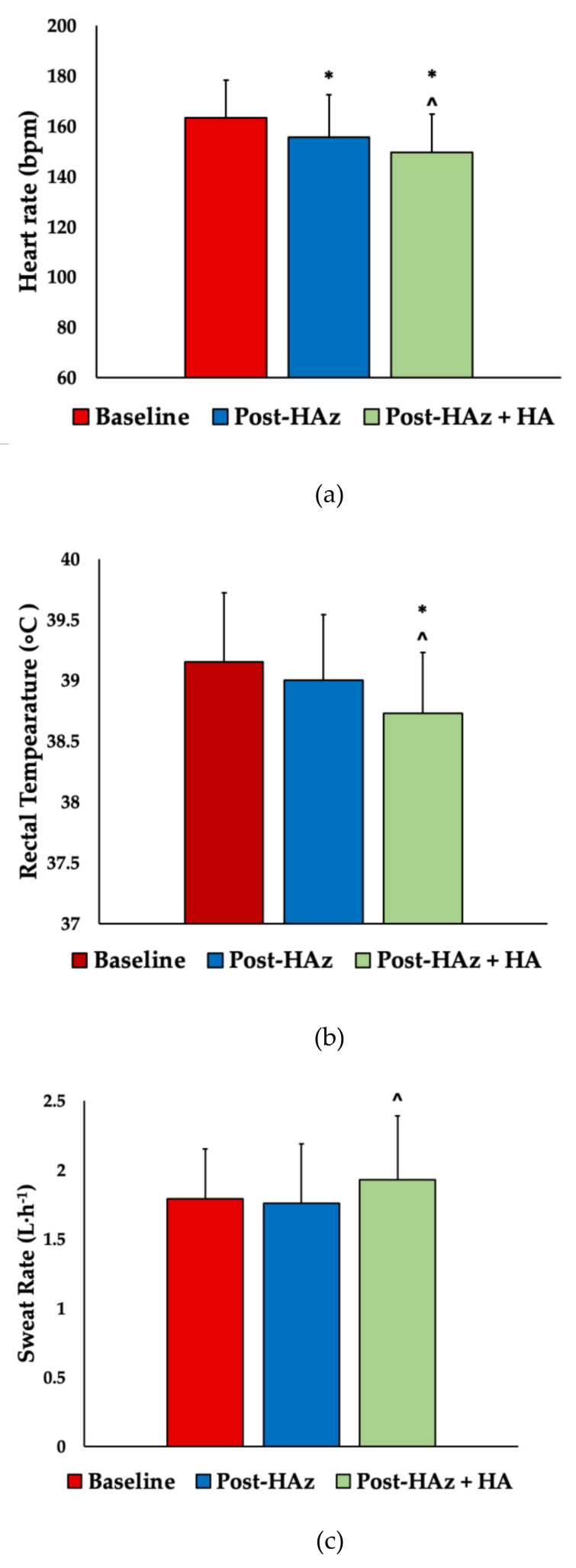
(**a**) Max heart rate, (**b**) max rectal temperature, and (**c**) sweat rate during 60 min of exercise in the heat during baseline, post-heat acclimatization (HAz), and post-heat heat acclimatization plus heat acclimation (HAz + HA). * Indicates statistical differences from baseline, *p* < 0.05. ˆ Indicates statistical differences from post HAz.

Pairwise comparisons demonstrated that average HR was significantly lower post-HAz (138 ± 14 bpm) compared to baseline (143 ± 12 bpm; *p* = 0.002; ES = 0.38) and in post-HAz+HA (134 ± 11 bpm) compared to baseline (*p* < 0.001; ES = 0.78). Additionally, average HR was significantly lower post-HAz+HA compared to post-HAz (*p* = 0.013; ES = 0.32). Max HR was significantly lower in post-HAz (155 ± 17 bpm; *p* = 0.002; ES = 0.50) and post-HAz+HA (150 ± 15 bpm; *p* < 0.001; ES = 0.87) compared to baseline (163 ± 15 bpm) (Figure 3). Max HR was significantly lower post-HAz+HA compared to post-HAz (*p* = 0.006; ES = 0.31).

### 3.2. T_rec_ and Delta T_rec_

Significant main effects were observed in average T_rec_ (*p* = 0.003), resting T_rec_ (*p* = 0.023), max T_rec_ (*p* < 0.001), and delta T_rec_ (*p* = 0.017). Pairwise comparisons demonstrated that average T_rec_ was significantly lower post-HAz+HA (38.03 ± 0.39 °C) compared to post-HAz (38.25 ± 0.42 °C; *p* = 0.009; ES = 0.54) and baseline (38.29 ± 0.37 °C; *p* = 0.005; ES = 0.68). No differences were found in baseline and post-HAz (*p* = 0.479). Resting T_rec_ was lower post-HAz+HA (37.00 ± 0.37 °C) compared to post-HAz (37.22 ± 0.37 °C; *p* = 0.016; ES = 0.60). No differences were found between baseline (37.19 ± 0.40 °C) and post-HAz (*p* = 0.577), and between baseline and post-HAz+HA (*p* = 0.067). Although approaching statistical differences, max T_rec_ was not different between baseline (39.15 ± 0.57 °C) and post-HAz (39.00 ± 0.54 °C, *p* = 0.059). However, post-HAz+HA max T_rec_ (38.73 ± 0.50 °C) was lower than baseline (*p* = 0.001; ES = 0.78) and post-HAz (*p* = 0.009; ES = 0.27) (Figure 3). Delta T_rec_ was significantly lower post-HAz (1.78 ± 0.45 °C; *p* = 0.025; ES = 0.34) and post-HAz+HA (1.73 ± 0.49 °C; *p* = 0.02; ES = 0.42) compared to baseline (1.96 ± 0.60 °C). There were no differences between post-HAz and post-HAz+HA (*p* = 0.337).

### 3.3. Skin Temperature and Sweat Rate

Significant main effects were observed in T_sk_ between trials (*p* < 0.001). T_sk_ was significantly lower post-HAz+HA (35.49 ± 0.62 °C) compared to post-HAz (35.86 ± 0.55 °C; *p* = 0.005; ES = 0.63) and compared to baseline (36.30 ± 0.46 °C; *p* < 0.001; ES = 1.48). T_sk_ was also significantly lower post-HAz compared to baseline (*p* = 0.001; ES = 0.87). Significant main effects were observed in SR between the trials (*p* = 0.029). SR was significantly higher post-HAz+HA (1.93 ± 0.47 L·h^−1^) compared to post-HAz (1.76 ± 0.43 L·h^−1^; *p* = 0.027; ES = 0.38). No SR differences were observed between baseline (1.79 ± 0.36 L·h^−1^) and post-HAz (*p* = 0.533), and between baseline and post-HAz+HA (*p* = 0.061), although the differences approached statistical significance (Figure 3).

### 3.4. Sweat Electrolyte Concentration

Significant main effects were observed in sweat [Na^+^] (*p* < 0.001) and [Cl^−^] (*p* < 0.001) were observed, however, no differences were observed in sweat [K^+^] (*p* = 0.208) between trials. Sweat [Na^+^] was lower post-HAz+HA (800.26 ± 227.23 mEq·L^−1^) compared to post-HAz (1067.17 ± 437.97 mEq·L^−1^; *p* = 0.001; ES = 0.77) and compared to baseline (1055.94 ± 386.34 mEq·L^−1^; *p* < 0.001; ES = 0.81). There were no observed differences in sweat [Na^+^] between baseline and post-HAz (*p* = 0.867). Sweat [Cl^−^] was lower post-HAz+HA (1186.67 ± 368.90 mEq·L^−1^) compared to post-HAz (1529.89 ± 648.49 mEq·L^−1^; *p* = 0.002; ES = 0.65) and compared to baseline (1565.56 ± 537.95 mEq·L^−1^; *p* < 0.001; ES = 0.82). There were no differences in sweat [Cl^−^] between baseline and post-HAz (*p* = 0.747).

### 3.5. Ratings of Perceived Exertion, Thermal Sensation, Thirst, and Fatigue

Significant main effects in RPE (*p* = 0.001), TS (*p* = 0.001), thirst, (*p* = 0.001), and fatigue (*p* = 0.027) were observed between tests. RPE was significantly lower post-HAz+HA (10 ± 2) compared to post-HAz (11 ± 2; *p* = 0.001; ES = 0.50) and compared to baseline (11 ± 2; *p* = 0.007; ES = 0.50). No differences in RPE were seen between baseline and post-HAz (*p* = 0.579). TS was significantly lower post-HAz+HA (5.1 ± 0.6) compared to post-HAz (5.5 ± 0.5; *p* = 0.003; ES = 0.72) and compared to baseline (5.6 ± 0.6; *p* = 0.001; ES = 0.83). There was no difference in TS reported between baseline and post-HAz (*p* = 0.633). Thirst was significantly lower post-HAz+HA (3 ± 1) compared to post-HAz (4 ± 1; *p* = 0.001; ES = 1.0) and compared to baseline (4 ± 1; *p* = 0.007; ES = 1.0).

There was no difference in thirst between baseline and post-HAz (*p* = 0.304). Perceived fatigue was lower post-HAz+HA (2 ± 1) compared to post-HAz (3 ± 1; *p* = 0.009; ES = 1.0), however no differences were observed between baseline (3 ± 1) and post-HAz (*p* = 0.137) and post-HAz+HA (*p* = 0.232).

## 4. Discussion

Athletes and coaches who live and train in milder summer climates may be under the impression that they have been completing HAz and are ready to compete in a hot environment, when in fact the thermal strain of their training was not enough to elicit the physiological adaptations that are needed in a more extreme climate. As a solution, our findings point to a novel, effective strategy that involves both HAz training during the summer months and a unique short-term HA protocol in an environmental chamber to elicit needed physiological and perceptual adaptations during exercise in the heat. Our unique protocol, which involved completing five days of HA following natural HAz, provides athletes and military personnel with a practical and effective strategy to optimize physiological outcomes during exercise in the heat. The five additional days of HA promoted adaptations that were not observed following HAz alone, indicating that HA was needed to optimize thermoregulatory benefits in the trial environmental conditions. Endurance athletes who trained throughout the summer months presented some physiological and perceptual improvements, however the short-term HA protocol following HAz elicited additional significant thermoregulatory benefits, including improvements in T_rec_, HR, and perceptual measures. Furthermore, we observed changes in SR, which is typically the latest adaptation to occur, giving merit to this method [4]. Additionally, this HZHA protocol is a novel approach that achieves high levels of thermal load that are often not achieved in traditional HA protocols.

Few studies have investigated the impacts of HAz on aerobically trained athletes. In contrast to the current findings, previous research did not report changes in HR, T_rec_, or sweat electrolyte concentration following summer training in a similar sample, although, the trial conditions were lower than the current study (30 °C T_amb_, 35% RH), which could have resulted in difficulty observing changes in physiological responses [21]. However, another study found that SR and sweat electrolyte concentration, but not HR, T_rec_, or T_sk_ improved following HAz in a soccer cohort [22]. These discrepancies are most likely due to the varying HAz protocols and a variety of other factors, such as duration, intensity, frequency, and time of day of training known to influence thermoregulatory responses [5,10].

While a review of short-term HA has been previously published, the novel protocol of HA in the current study produced meaningful changes that expands the current HA literature. Two reasons this specific protocol was unique was that; (1) it followed HAz, and (2) it utilized the HZHA approach. A study by Daanen et al. recently examined a two-part HA protocol that involved participants completing nine consecutive days in moderate conditions (35 °C T_amb_, 29% RH) followed by three days of severe conditions (41 °C T_amb_, 33% RH) [12]. The author’s conclusion was that a two-stage acclimation program did not result in enhanced physiological adaptations and that the short length of exposure (3 days) to the severe environment may have contributed to these findings. This hypothesis is strengthened with the findings from this study, as a longer (5-day) HA protocol following HAz elicited further adaptations.

While improvements in several physiological variables were seen from baseline to post-HAz (commonly classified as long-term), improvements were also observed from short-term HA. A review of short-term HA protocols states that HR lowers by approximately 6–10%, and resting and exercise T_rec_ lowers by 0.2 °C following short-term HA [23]. These cardiovascular responses are consistent with our 9% improvement of HR following HAz and an additional 4% following HA. These improvements in HR can likely be attributed to plasma volume expansion that often results from HA, which would ultimately result in a more efficient stroke volume and improved maintenance of cardiac output [24]. Max T_rec_ improved by 0.3 °C in the present study following HA and 0.4 °C following HA, which is slightly higher than previously reported data [23].

Unlike many previous studies, the SR of the participants in this study increased by 9% following HA [7]. Few studies have observed SR improvements following short-term HA [23], however, those that did demonstrate improvement in this thermoregulatory benefit were completed in some of the more extreme environments (38–40 °C T_amb_, 12–60% RH) with intense protocols [22] or with the additional stress of equipment [25,26]. Another common response following HA is a reduced sweat electrolyte concentration [27]. Specifically, reduced [Na^+^] and [Cl^−^], most often collected through the use of regional sweat patches, has been observed in previous research in as soon as two days into a HA protocol [28]. In the current study, HAz did not result in improvements in [Na^+^] or [Cl^−^] and this is most likely due to the mild environmental conditions observed throughout this training period. In contrast, the extreme environmental conditions during HA resulted in significantly lower [Na^+^] and [Cl^−^]. As expected, no changes were observed in [K^+^]. In addition to physiological improvements, perceptual improvements were also observed from HA but not HAz. In this highly aerobically trained population, improvements are often more difficult to observe. We propose that the mechanism behind the physiological and perceptual improvements following HA in the present study is a direct result of the HZHA protocol used.

While there is clear evidence that HA results in several physiological adaptations, the wide range of the magnitude and time course of these physiological responses reported in previous literature could be due to the wide range of variability in program designs. The improvements demonstrated from HZHA and the concept of safely maximizing the area under the T_rec_ curve could help explain some of the discrepancies seen in previous HA literature. This concept is based on the simple, yet thorough, overload training principle, in which the body adapts to the stresses it is placed under [29]. Unlike previously reported HA protocols, HZHA may be effective at eliciting greater positive physiological adaptations because this protocol leads to elevated internal body temperature and an increased sweating response.

One limitation of the present study was that there was no control group to account for a training effect. While a control group could have improved this study design, the high aerobic fitness of the participants in this study and the fact that no changes in VO_2peak_ or vVO_2peak_ were observed over time reduces the chance that training alone would have impacted these results. In addition, it is unclear from the present study design if HAz induced preliminary and incomplete adaptations prior to HA, which could have influenced the results. Another limitation of the current study is that internal body temperature was not captured throughout HAz. Future research should aim to examine internal body temperature periodically throughout HAz to assess the internal thermal load during summer training, which would allow researchers to better understand the AUC that occurs during this time. Another limitation to the present study is that the physiological and perceptual changes following HAz were small to moderate, most likely due to the location of training (New England, USA) and, therefore, relatively mild environmental conditions. Future research should look to complete this study in more extreme climates. Another limitation, due to the nature of this study, is that the days between testing were not identical across participants, although, the variations were limited and likely did not impact these outcomes. Finally, a limitation to this study is that female athletes were not included due to the impacts that menstrual cycle may have on internal body temperature. Future research should strongly consider including female athletes.

## 5. Conclusions

HAz resulted in some valuable improvements to physiological outcomes that indicate positive thermoregulatory benefits and a short-term HZHA protocol led to additional significant benefits. This novel training approach is an efficient and effective method to optimize performance in the heat, and may help decrease the health risks associated with intense exercise in the heat. Following the combination of HAz and HA, average HR was 8 bpm lower (moderate effect) and maximal HR was 14 bpm lower (large effect) compared to baseline. Average T_rec_ was 0.26 °C lower (moderate effect) and maximal T_rec_ was 0.42 °C lower (moderate effect) compared to baseline. This effective method of obtaining the benefits of HAz and HA is potentially useful for athletes, military personnel, and physically active individuals who are at risk for exertional heat illness and are aiming for peak performance during exercise in the heat.

## Figures and Tables

**Table 1:** Self-directed summer training (HAz) and environmental data. Data are describing averages for each training session. Data are reported as mean ± standard deviation (M ± SD).

Exercise Type (# of Sessions)	Distance (km)	Heart Rate (bpm)	Duration (min)	Heat Index (°C)	WBGT (°C)	Time of Day (hh:mm)
Outdoor Running (*n* = 1692)	10.28 ± 8.43	140 ± 15	56.38 ± 72.66	29.89 ± 2.42	22.31 ± 4.23	12:14 ± 4:42
Outdoor Cycling (*n* = 364)	32.74 ± 26.21	128 ± 16	91.67 ± 69.27	30.17 ± 2.41	23.68 ± 3.96	13:12 ± 3:52
Multi-Sport (*n* = 18)	27.88 ± 15.43	125 ± 6	90.71 ± 31.78	31.32 ± 1.51	22.03 ± 6.20	11:11 ± 4:33
Hiking (*n* = 19)	8.50 ± 8.95	94 ± 18	161.58 ± 170.58	30.77 ± 4.61	19.39 ± 6.84	11:50 ± 3:30

**Table 2:** Physiological variables collected throughout the 5-day heat acclimation protocol. Data are reported as mean ± standard deviation.

-	HA^#1^	HA^#2^	HA^#3^	HA^#4^	HA^#5^	Overall
Total Session						
Duration (min)	82 ± 6	81 ± 6	85 ± 6	83 ± 8	83 ± 8	83 ± 5
Average HR (bpm)	137 ± 13	132 ± 14	132 ± 11	130 ± 12	129 ± 12	132 ± 11
Average T_rec_ (°C)	38.85 ± 0.42	38.93 ± 0.31	38.81 ± 0.38	38.80 ± 0.30	38.78 ± 0.31	38.83 ± 0.25
Max HR (bpm)	165 ± 13	164 ± 11	164 ± 13	162 ± 14	161 ± 15	163 ± 11
Max T_rec_ (°C)	39.63 ± 0.34	39.65 ± 0.28	39.46 ± 0.30	39.50 ± 0.28	39.48 ± 0.29	39.55 ± 0.15
AUC (°C·h^−1^)	52 ± 4	51 ± 4	51 ± 3	52 ± 4	52 ± 5	52 ± 4
Perceived Exertion	10 ± 2	10 ± 2	10 ± 2	10 ± 2	10 ± 2	10 ± 2
Thermal Sensation	6.0 ± 1.0	6.0 ± 1.0	6.0 ± 1.0	6.0 ± 1.0	6.0 ± 0.5	6.0 ± 1.0
Fatigue	4 ± 2	3 ± 2	4 ± 2	3 ± 2	3 ± 2	3 ± 2
Sweat Volume (L)	2.40 ± 0.63	2.47 ± 0.59	2.72 ± 0.60	2.74 ± 0.56	2.77 ± 0.81	2.62 ± 0.52
Session after 38.5 °C *						
Average T_rec_ (°C)	39.16 ± 0.42	39.24 ± 0.22	39.16 ± 0.36	39.16 ± 0.30	39.11 ± 0.22	39.17 ± 0.17
Average HR (bpm)	138 ± 14	132 ± 14	131 ± 14	131 ± 14	128 ± 12	132 ± 12
AUC (°C·h^−1^)	46 ± 16	46 ± 12	42 ± 16	42 ± 15	38 ± 10	43 ± 13

* The heat acclimation protocol called for 60 min above 38.5 °C; HA^#x^: day of heat acclimation; T_rec_: rectal temperature; HR: heart rate; AUC: area under the curve; data are presented as mean ± standard deviation.

**Table 3:** Physiological outcomes from heat acclimatization, heat acclimation, and the combination of heat acclimatization and heat acclimation.

Physiological Outcome	Baseline vs. Post-HAz			Post-HAz vs. Post-HAz+HA			Baseline vs. Post-HAz+HA		
	MD ± SE	ES	*p*-Value	MD ± SE	ES	*p*-Value	MD ± SE	ES	*p*-Value
Average Heart Rate (bpm)	−5 ± 1	0.36	0.002 *	−4 ± 3	0.29	0.013 *	−8 ± 2	0.71	<0.001 *
Max Heart Rate (bpm)	−8 ± 2	0.47	0.002 *	−6 ± 2	0.36	0.06 *	−14 ± 2	0.90	<0.001 *
Average T_rec_ (°C)	−0.04 ± 0.01	0.08	0.479	−0.22 ± 0.08	0.54	0.009 *	−0.26 ± 0.08	0.68	0.005 *
Max T_rec_ (°C)	−0.15 ± 0.07	0.27	0.059	−0.27 ± 0.10	0.52	0.009 *	−0.42 ± 0.11	0.78	0.001 *
Minimum T_rec_ (°C)	0.04 ± 0.06	0.08	0.577	−0.22 ± 0.10	0.59	0.016 *	−0.18 ± 0.10	0.49	0.067
Delta T_rec_ (°C)	−0.18 ± 0.08	0.34	0.025 *	−0.05 ± 0.05	0.11	0.337	−0.23 ± 0.09	0.42	0.020 *
Average T_SK_ (°C)	−0.45 ± 0.11	0.87	0.001 *	−0.37 ± 0.11	0.63	0.005 *	−0.81 ± 0.12	1.48	<0.001 *
Sweat Rate (L·h^−1^)	−0.03 ± 0.05	0.08	0.533	0.16 ± 0.07	0.36	0.027 *	0.13 ± 0.07	0.31	0.061

Baseline: Unacclimated; post-HAz: post-heat acclimatization; post-HAz+HA: post-heat acclimatization + heat acclimation; negative values indicate the later test is lower than the earlier test; positive values indicate later test is higher than earlier test; data are presented as mean difference ± standard error and effect size; * indicates statistical significance, *p* < 0.05.

## Data Availability

The data for the current study is available in this manuscript.
